# County-level associations between passively collected walking and bicycling and self-reported nonoccupational physical activity data

**DOI:** 10.1371/journal.pone.0359535

**Published:** 2026-10-06

**Authors:** Tiffany J. Chen, Miriam E. Van Dyke, Graycie W. Soto, Bryant J. Webber, Kelly Fletcher, Sarah Rockhill, Michael D. Garber, Akimi Smith, Geoffrey P. Whitfield

**Affiliations:** 1 Division of Nutrition, Physical Activity, and Obesity, National Center for Chronic Disease Prevention and Health Promotion, Centers for Disease Control and Prevention, Atlanta, Georgia, United States of America; 2 McKing Consulting Corporation, Atlanta, Georgia, United States of America; 3 Oak Ridge Institute for Science and Education Research Participation Program, Oak Ridge, Tennessee, United States of America; 4 Epidemic Intelligence Service, Centers for Disease Control and Prevention, Atlanta, Georgia, United States of America; 5 Geospatial Research, Analysis, and Services Program, Centers for Disease Control and Prevention, Atlanta, Georgia, United States of America; 6 Scripps Institution of Oceanography, University of California San Diego, San Diego, California, United States of America; 7 Office on Smoking and Health, National Center for Chronic Disease Prevention and Health Promotion, Centers for Disease Control and Prevention, Atlanta, Georgia, United States of America; Shanghai University, CHINA

## Abstract

Passively collected location-based services data on walking and bicycling trips could potentially complement traditional public health surveillance systems as related but distinct sources for physical activity data. Starting with a sample of 298 counties, we explored county-level associations between 2019 StreetLight location-based services measures and 2017 and 2019 Behavioral Risk Factor Surveillance System self-reported nonoccupational physical activity measures using Spearman’s rank correlation coefficients (rho), following Cohen’s interpretation (low: < 0.3; moderate: 0.3 to <0.5; strong: ≥ 0.5). Then, we explored stratified associations for the two strongest correlated StreetLight–Behavioral Risk Factor Surveillance System pairs of measures to understand how these associations differed by county characteristics. Of 72 StreetLight–Behavioral Risk Factor Surveillance System measure combinations, 4 pairs were moderately correlated (rho 0.3 to <0.5). The remaining pairs had low correlations (rho < 0.3). Select stratified measure pairs had strong correlations (rho ≥ 0.5) for medium and small metro counties and counties in the middle tertile of social vulnerability. Location-based services measures and Behavioral Risk Factor Surveillance System physical activity measures mostly showed limited convergent validity. The usefulness of location-based services data for representing nonoccupational physical activity may be context-sensitive to certain types of counties, where the constructs may have more in common.

## Introduction

Physical activity (PA) provides important health benefits for everyone, such as reduced risk of chronic disease, feelings of anxiety, and depression. It also improves sleep, cognitive function, and body weight and composition [1]. For adults, the *Physical Activity Guidelines for Americans,* second edition, recommends at least 150 minutes of moderate-intensity or 75 minutes of vigorous-intensity aerobic PA each week (or an equivalent combination), spread throughout the week (aerobic guideline), and moderate or greater intensity muscle-strengthening activities at least twice a week (muscle-strengthening guideline) [1]. Traditionally, adult PA has been assessed nationally by public health or other surveillance systems using surveys [2]. While these surveillance systems provide valuable national-level data on PA, they are often unable to provide reliable data at granular geographic resolutions (i.e., local-level estimates) [3], require longer time periods for data availability due to the infrequency of data collection or subsequent processing and reporting after collection, are subject to bias related to self-reporting behaviors (e.g., social desirability and other recall biases), and suffer from declining survey response rates [2].

Advances in mobile-device technologies provide both a challenge to traditional PA surveys (e.g., individuals screening their phone calls) and an emerging opportunity to use location-based services (LBS) data to improve surveillance of aerobic PA by offsetting some of the limitations of traditional public health data sources [2]. LBS data consist of geographic location information collected passively via mobile apps. Commercial vendors such as StreetLight Data, Inc. (henceforth, “StreetLight”) provide aggregated, anonymized travel data via proprietary algorithms that categorize LBS data into trips with assigned modes, including walking and bicycling [4].

Emerging evidence suggests LBS data may be a useful source of information about population PA that could be more representative than other location-based app data subject to self-selection bias (e.g., fitness apps with users biased towards PA) [5,6]. LBS data were used to measure changes in utilitarian and leisure walking during the COVID-19 pandemic [7]. However, there are limited studies comparing LBS data with data from traditional public health PA surveillance systems. In one study, StreetLight LBS data demonstrated moderate to strong associations at the county level with active commuting behaviors measured by the American Community Survey (ACS) [8]. Those findings suggest LBS data could complement traditional surveillance data with timelier and more geographically precise estimates of active transportation. StreetLight LBS measures have not been compared with measures capturing other domains of PA (e.g., domestic or leisure-time) in a national public health surveillance system, such as the Behavioral Risk Factor Surveillance System (BRFSS) that assesses nonoccupational PA. A precedent for exploring convergent validity of distinct but related PA assessment constructs has been set by comparisons between objective accelerometer data and self-reported survey data [9], and exploring how human movement data such as StreetLight LBS measures compare to self-reported BRFSS nonoccupational PA survey measures has the potential to show population-level correlation.

The objective of our study was to explore county-level convergent validity between various LBS measures of walking and bicycling from StreetLight and self-reported survey measures of nonoccupational PA from BRFSS. The results of this study may inform future use of LBS data as a complement to traditional PA surveillance systems. We first aimed to identify which combinations of StreetLight measures and BRFSS measures were most strongly associated. For the pairs with the strongest associations, we next aimed to assess these associations across selected county-level sociodemographic and geographic characteristics.

## Methods

### Sample design

We obtained aggregated, anonymized walking and bicycling trip measures from StreetLight for a purposeful sample of 298 counties from the contiguous United States. This sample represented all regions and urbanicity levels, first selecting the 68 most urban counties per the 2013 National Center for Health Statistics (NCHS) urban-rural classification scheme (largely categorized by population size of the county’s metropolitan statistical area) [10], and then randomly sampling an additional 230 counties stratified by both the remaining 5 levels of the NCHS classification scheme and the 4 Census regions. Detailed sampling methodology is described elsewhere [8].

### Walking and bicycling data from StreetLight

At the time of data purchase, StreetLight used LBS data from multiple mobile device applications (e.g., weather or dating apps) that collect anonymous user locations when an app is in use or running in the background processes of the device [11]. StreetLight combined these data with data from other sources, including fitness applications in which mode is specified, and validated pedestrian and bicycle counts from permanent counters. Then, StreetLight used a proprietary algorithm to detect trips and assign the most likely trip mode using estimates of speed (i.e., from locations over time as a device is moving) and other trip attributes. Detailed methods are available from StreetLight [11]. StreetLight does not process, use, or distribute personally identifiable information in their products. Their sources collect location-based data on an “opt-in” basis and are expected to remove personally identifiable information from location records, then further de-identify remaining information before providing to StreetLight. After StreetLight transforms these data algorithmically, they produce metrics in aggregated composite groups. Due to insufficient sample sizes, StreetLight suppressed some bicycling trip metrics, which resulted in decreased county sample size for select analyses of county residents that include bicycling (i.e., a maximum of 4 sampled counties with missing bicycling data, for a minimum sample size of 238 counties from bicycling suppression).

Using LBS data from 2019, Streetlight produced a dataset that included average daily trip counts and average trip durations in seconds, for any trip starting in the sampled counties (S1 Dataset). Trip characteristics included mode (walking or bicycling), day type (all days, weekdays, or weekends), and traveler type (trips by any person or trips restricted to county residents). Streetlight estimated block group “home” locations based on where the device most often spent the night and “work” locations based on where the device most often spent daytime hours [11]. Proportion estimates for three trip purposes were available among trips by the “any person” traveler type: home-based work trips (i.e., home-to-work or work-to-home); home-based other trips (i.e., to or from home, but not home-based work); and not home-based trips (i.e., not to or from home) (Fig 1). We also refer to home-based other and not home-based trips as “non-commute” trips. We created various combinations of these trip attributes that resulted in the following StreetLight trip measures for walking only and walking and bicycling combined, each for average daily trips and average durations of trips:

**Fig 1:**
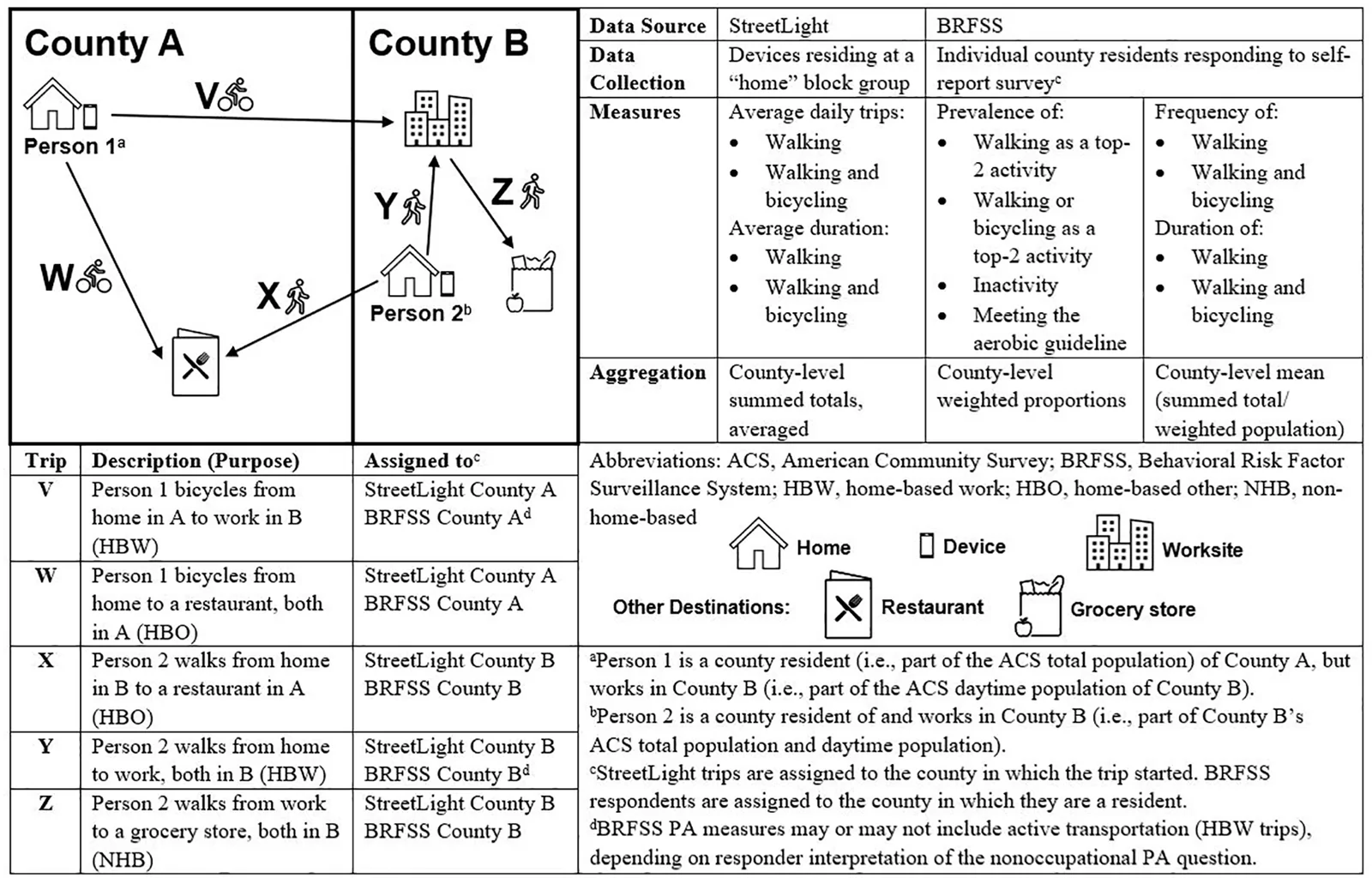
Data collection, measures, aggregation, and trip purpose with examples by data source.

- Taken on all days by any person for all purposes
- Taken on weekdays by any person for all purposes
- Taken on weekends by any person for all purposes
- Taken on all days by any person for non-commute purposes
- Taken on weekdays by any person for non-commute purposes
- Taken on weekends by any person for non-commute purposes
- Taken on all days by county residents for all purposes
- Taken on weekdays by county residents for all purposes
- Taken on weekends by county residents for all purposes

### Nonoccupational physical activity data from the Behavioral Risk Factor Surveillance System

The BRFSS is a national, state-based surveillance system that uses telephone surveys with a standardized questionnaire to collect health-related data on civilian, noninstitutionalized U.S. adults aged ≥18 years. PA data were collected by BRFSS biennially during odd years. We accessed restricted-use county identifiers and combined data from 2017 and 2019 to increase the number of counties with enough sample size to produce stable estimates. Survey response rates were 45.9% overall in 2017 and 49.4% overall in 2019 [12,13]. The BRFSS protocol was approved by the CDC institutional review board and was determined to be exempt. Upon introduction to the survey, respondents are informed of the purpose of the survey, who is conducting the survey and a contact telephone number, and the following: 1) they will not be asked for information that can identify them, 2) information they provide will not be connected to any personal information, 3) they do not have to answer any question they do not want to, and 4) they can end the interview at any time. BRFSS data are publicly available and can be accessed at https://www.cdc.gov/brfss/annual_data/annual_data.htm.

### Data collection instruments

BRFSS assesses self-reported nonoccupational PA by asking, “During the past month, other than your regular job, did you participate in any physical activities or exercises such as running, calisthenics, golf, gardening, or walking for exercise?” This question includes domestic (i.e., housework or chores), transportation or utilitarian (i.e., to go somewhere), and leisure-time (i.e., recreational, during free time) domains of PA [14], though BRFSS PA assessments have generally been used as leisure-time PA assessments [15]. Response options were “Yes,” “No,” “Don’t know/Not sure,” and “Refused”. Respondents who answered “Yes” were then asked about their past-month PA types for the top 2 activities that they spent the most time doing (i.e., 75 activities as response options, such as walking, bicycling, gardening, and running), past-month PA frequencies for their top 2 activities (e.g., times per week or month), and durations of these PA episodes for their top 2 activities (e.g., minutes or hours) [16,17].

### Measures

Using individual-level responses to the instruments above, we derived 8 nonoccupational PA variables summarized at county level. First, respondents who selected “No” to participating in any past-month physical activities or exercises were categorized as physically inactive, and “Don’t know/Not sure” and “Refused” were considered missing, resulting in an inactivity prevalence measure:

- Prevalence of inactivity among all adults

Second, for responses to activities, we categorized selections of “walking,” “hiking - cross-country,” and “backpacking” as walking; there was only one option for bicycling. Inactive respondents were categorized as not walking and not bicycling. Top-2 activity variables were created from responses that use the first and/or second top activities of walking only, and walking or bicycling. Bicycling was too rare to be analyzed in isolation. Using these variables, we calculated 2 prevalence measures:

- Prevalence of all adults reporting walking as a top-2 activity in the past month
- Prevalence of all adults reporting walking or bicycling as a top-2 activity in the past month

Third, we summed reported frequencies of episodes of walking only and walking and bicycling, for which we calculated 2 measures:

- Mean frequency of walking in a week among all adults reporting walking as a top-2 activity in the past month
- Mean frequency of walking and bicycling in a week among all adults reporting walking or bicycling as a top-2 activity in the past month

Fourth, we summed reported durations of walking only and walking and bicycling, for which we calculated 2 measures:

- Mean duration of walking minutes per instance in a week among all adults reporting walking as a top-2 activity in the past month
- Mean duration of walking and bicycling minutes per instance in a week among all adults reporting walking or bicycling as a top-2 activity in the past month

Total moderate-intensity equivalent minutes of PA per week was calculated using frequency per week of top-2 activities, combined top-2 activity durations, and vigorous-equivalent minutes (i.e., 2 minutes of moderate activity per 1 minute of vigorous activity) [18,19]. Respondents who reported doing ≥150 minutes of moderate-intensity equivalent PA in a week were classified as meeting the aerobic guideline; those reporting less were classified as not meeting the aerobic guideline. “Don’t know/Not sure/Refused” or missing responses to inactivity, frequency, or duration were considered missing. This resulted in a prevalence measure for meeting the aerobic guideline:

- Prevalence of meeting the aerobic guideline among all adults

### Suppression

From the sample of 298 counties, 40 were excluded due to insufficient sample size in BRFSS (<50 respondents for 2017 and 2019 combined). From this maximum of 258 counties, we excluded additional counties based on each BRFSS measure; unstable prevalence estimates were suppressed according to the NCHS data presentation standards for proportions [20], and frequency or duration means with a relative standard error of >30% were suppressed. These criteria resulted in 16 counties excluded for prevalence of inactivity (n = 242 counties), 20 counties excluded for mean duration of walking minutes per instance in a week (n = 238 counties), 18 counties excluded for mean duration of walking and bicycling minutes per instance in a week (n = 240 counties), and 23 counties excluded for prevalence of meeting the aerobic guideline (n = 235 counties). Nineteen counties were excluded for prevalence of all adults reporting walking as a top-2 activity and prevalence of all adults reporting walking or bicycling as a top-2 activity (n = 239 counties for both measures); five counties were excluded for mean frequency of walking in a week and mean frequency of walking and bicycling in a week (n = 253 counties for both measures).

### County population and characteristics data from the American Community Survey

The ACS is an annual, representative nationwide survey administered by the U.S. Census Bureau using internet or paper questionnaires. It samples approximately 3.5 million households by address, and each selected household completes the questionnaire for all household members to provide social, economic, housing, and demographic characteristics information at several geographic levels for the U.S. population. ACS data are used to produce 1-year and 5-year estimates [21]; methodological details on these estimates are presented elsewhere [8]. We used 5-year estimates (2015–2019) of county populations as denominators to calculate rates and obtained county-level sociodemographic and geographic information to stratify results. ACS data are publicly available and can be accessed at https://www.census.gov/programs-surveys/acs/data.html.

To calculate average trip count rates and average trip duration rates (per 1,000 people), we divided StreetLight walking and walking and bicycling numerator measures by the most appropriate ACS county population denominator to match the StreetLight traveler type for each measure. For StreetLight trip measures that included combined residents and non-residents (i.e., “any person” in the county), we calculated a trip rate using the ACS denominator of commuter-adjusted daytime population (henceforth, “daytime population”). A county’s daytime population is an estimate of the number of people present in the county during normal business hours; it can be calculated by adding the total workers working in the county to the county’s total resident population and subtracting the total workers living in the county. For StreetLight trip measures that included county residents only, we calculated a trip rate using the ACS denominator of county resident population. A county’s resident population refers to people who reside in the county and are presumably present in the county after normal business hours, in the evening and nighttime [22].

We stratified results by county characteristics from ACS. Geographic and population stratifiers were U.S. Census region (Midwest, Northeast, South, West), urbanicity (large central metro, large fringe metro, medium metro, small metro, and micropolitan/non-core) [10], population, and population density (i.e., county population per county square mile [from the U.S. Census Bureau]). Sociodemographic stratifiers were median age, race/ethnicity (i.e., percent of the county population self-reported as non-Hispanic White [White], non-Hispanic Black [Black], or Hispanic or Latino/a [Hispanic]), education (i.e., percent of the county population with at least some college education), poverty status (i.e., percent of the county population that was below the federal poverty level in the past 12 months), and median income. Lastly, we stratified by the CDC/Agency for Toxic Substances and Disease Registry 2018 Social Vulnerability Index (SVI), which ranks communities based on measures of vulnerability (e.g., socioeconomic status, household characteristics, race and ethnicity, and housing and transportation) [23].

### Statistical analysis

Using the most heavily suppressed sample of counties in our analysis (n = 235), we described these counties by the characteristics above and present comparable statistics for all U.S. counties and county equivalent areas (i.e., places comparable to counties, such as parishes) (N = 3,142). County descriptive statistics were stratified by median for population and by tertiles for the remaining characteristics.

We assessed associations between county-level StreetLight LBS measures (by combinations of mode, trip frequency or duration, day type, traveler type, trip purpose, and population denominator) and BRFSS nonoccupational PA measures (by various measures of activity, mode, prevalence or mean frequency or duration) using Spearman’s rank correlation coefficients (rho) following Cohen’s interpretation (low: < 0.3; moderate: 0.3 to <0.5; strong: ≥ 0.5). To understand how associations differ by county characteristics, we identified the two StreetLight–BRFSS pairs of measures with the strongest correlation coefficients and repeated the analysis, stratifying by the county characteristics described above. For each StreetLight and BRFSS measure, we calculated the median and interquartile range of the stratified county estimates. Alpha level was p = 0.05. Analyses were conducted using SAS-callable SUDAAN (version 11.0; Research Triangle Institute, Research Triangle Park, North Carolina) and BRFSS analyses accounted for survey weights and complex survey design. Scatterplots were created using R (version 4.3.2; R Foundation for Statistical Computing, Vienna, Austria).

### Regulatory information

This activity was reviewed and approved by CDC Human Subject Contact and Paperwork Reduction Act reviewers, deemed as public health surveillance and not research, and was conducted consistent with applicable federal law and CDC policy (see, e.g., 45 C.F.R. part 46.102(l)(2), 21 C.F.R. part 56; 42 U.S.C. §241(d); 5 U.S.C. §552a; 44 U.S.C. §3501 et seq.). CDC did not receive any personally identifiable information from StreetLight, the Behavioral Risk Factor Surveillance System, or the American Community Survey, and authors did not have access to information that could identify individuals at any point. All data were fully anonymized before they were obtained and used for secondary data analyses. Data were accessed December 8, 2021 through October 10, 2024 for the purposes of this study.

## Results

### Sampled county characteristics

Our sampled counties represent 7.5% of all 3,142 U.S. counties and county equivalents, but 42.7% of the total U.S. population (138.7 million of 324.7 million people, Table 1), which is expected in an oversample of populous urban counties. Our counties were also more urbanized than U.S. counties overall, overrepresenting large central metro, large fringe metro, and medium metro counties (28.9%, 27.2%, and 23.0% versus 2.2%, 11.7%, and 11.8% overall, respectively) and underrepresenting micropolitan/non-core counties (12.3% versus 62.9% overall). Additionally, our sample skews towards greater racial and ethnic diversity and higher education and income than the census of all U.S. counties. Differences in the distribution of SVI were smaller than for other characteristics (Table 1).

**Table 1:** Characteristics of all U.S. counties (ACS, 2015-2019) and overlapping StreetLight (2019) and BRFSS (2017 and 2019) sampled counties from BRFSS prevalence measure of meeting the aerobic physical activity guideline^a^.

Characteristic^b^	United States^c^	StreetLight/BRFSS Sampled Counties
No. counties	3142	235
Total population, millions	324.7	138.7
Census region		
Midwest	1,055 (33.6%)	51 (21.7%)
Northeast	217 (6.9%)	54 (23.0%)
South	1,422 (45.3%)	81 (34.5%)
West	448 (14.3%)	49 (20.9%)
Urbanicity^d^		
Large central metro	68 (2.2%)	68 (28.9%)
Large fringe metro	368 (11.7%)	64 (27.2%)
Medium metro	372 (11.8%)	54 (23.0%)
Small metro	358 (11.4%)	20 (8.5%)
Micropolitan/Non-core	1,976 (62.9%)	29 (12.3%)
Population		
≤ median (≤ 233,464)^e^	2,861 (91.1%)	118 (50.2%)
> median (>233,464)	281 (8.9%)	117 (49.8%)
Population density^f^		
Tertile 1 (< 219.4)	2,621 (83.4%)	78 (33.2%)
Tertile 2 (219.4–1,014.3)	372 (11.8%)	78 (33.2%)
Tertile 3 (> 1,014.3)	149 (4.7%)	79 (33.6%)
Median age, years		
Tertile 1 (< 36.5)	504 (16.0%)	77 (32.8%)
Tertile 2 (36.5–40.3)	784 (25.0%)	82 (34.9%)
Tertile 3 (> 40.3)	1,854 (59.0%)	76 (32.3%)
White race		
Tertile 1 (< 57.3%)	557 (17.7%)	78 (33.2%)
Tertile 2 (57.3% – 79.9%)	818 (26.0%)	77 (32.8%)
Tertile 3 (> 79.9%)	1,767 (56.2%)	80 (34.0%)
Black race		
Tertile 1 (< 3.2%)	1,779 (56.6%)	78 (33.2%)
Tertile 2 (3.2% – 12.9%)	696 (22.2%)	78 (33.2%)
Tertile 3 (> 12.9%)	667 (21.2%)	79 (33.6%)
Hispanic ethnicity		
Tertile 1 (< 5.4%)	1,839 (58.5%)	80 (34.0%)
Tertile 2 (5.4–13.0%)	710 (22.6%)	77 (32.8%)
Tertile 3 (> 13.0%)	593 (18.9%)	78 (33.2%)
Some college or more		
Tertile 1 (< 56.6%)	1,992 (63.4%)	78 (33.2%)
Tertile 2 (56.6–65.3%)	785 (25.0%)	79 (33.6%)
Tertile 3 (> 65.3%)	365 (11.6%)	78 (33.2%)
Below federal poverty level		
Tertile 1 (< 10.6%)	784 (25.0%)	78 (33.2%)
Tertile 2 (10.6–14.9%)	938 (29.8%)	79 (33.6%)
Tertile 3 (> 14.9%)	1,420 (45.2%)	78 (33.2%)
Median income		
Tertile 1 (<$55,630)	2,017 (64.2%)	78 (33.2%)
Tertile 2 ($55,630–68,666)	770 (24.5%)	79 (33.6%)
Tertile 3 (> $68,666)	355 (11.3%)	78 (33.2%)
CDC SVI percentile^g,h^		
Tertile 1 (< 0.33)	1,038 (33.0%)	79 (33.6%)
Tertile 2 (0.33–0.62)	909 (28.9%)	74 (31.5%)
Tertile 3 (> 0.62)	1,194 (38.0%)	82 (34.9%)

Abbreviations: ACS, American Community Survey; BRFSS, Behavioral Risk Factor Surveillance System; CDC SVI, Centers for Disease Control and Prevention social vulnerability index

^a^Meeting the aerobic physical activity guideline is defined as ≥150 minutes/week moderate intensity, ≥ 75 minutes/week vigorous intensity, or an equivalent combination of physical activity per week, according to the *Physical Activity Guidelines for Americans*, second edition

^b^All county characteristics data from ACS (2015–2019) except where notated

^c^All counties and county equivalents in the 50 states and District of Columbia in 2010

^d^From the 2013 National Center for Health Statistics Urban-Rural Classification Scheme

^e^Median population and all tertiles calculated using ACS data (2015–2019) from the 235-county sample for the BRFSS prevalence measure of meeting the aerobic physical activity guideline

^f^Defined as persons per square mile

^g^From the 2018 CDC SVI overall vulnerability index, with tertile 3 representing most vulnerable (for all U.S. counties, n = 3,141)

^h^County distributions were statistically different between the United States and the StreetLight/BRFSS sampled counties for all characteristics (chi square p < .05), except for by CDC SVI percentile.

### Correlations between StreetLight LBS measures–BRFSS nonoccupational PA measures

Of the 72 StreetLight–BRFSS measure combinations, 68 pairs had low correlations (<0.3) and 4 pairs had moderate correlations (0.3 to <0.5, Tables 2-6). When comparing walking and bicycling trip rates per 1,000 people to prevalence estimates of inactivity and meeting the aerobic guideline, all correlation coefficients were <0.3 except for one moderate correlation: the weekend, any-person, non-commute walking and bicycling trip rate and the prevalence of adults who met the aerobic guideline (rho = 0.32, p < .0001, Table 2). This was the most strongly correlated pair of the 72 StreetLight–BRFSS measure combinations.

**Table 2:** County rank correlations between StreetLight measures of walking and bicycling trips and BRFSS prevalence measures of inactivity and meeting the aerobic guideline^a^.

StreetLight Walking and Bicycling Trips per 1K Population, 2019				BRFSS Physical Activity Measures, 2017 and 2019	
Day	Traveler Type	Purpose	Denominator	Percent of all adults who are physically inactive	Percent of all adults who meet the aerobic physical activity guideline^d^
All	Any	All	DP	−0.10^b^	**0.25**
Weekdays	Any	All	DP	−0.11^b^	**0.24**
Weekends	Any	All	DP	−0.09^b^	**0.27**
All	Any	HBO or NHB	DP	**−0.18** ^b^	**0.29**
Weekdays	Any	HBO or NHB	DP	**−0.18** ^b^	**0.26**
Weekends	Any	HBO or NHB	DP	**−0.18** ^b^	**0.32**
All	Residents	All	Residents	**−0.14** ^b^	**0.23**
Weekdays	Residents	All	Residents	**−0.14** ^b^	**0.22**
Weekends	Residents	All	Residents	**−0.14** ^c^	**0.27**

Abbreviations: BRFSS, Behavioral Risk Factor Surveillance System; DP, daytime population; HBO, home-based other; NHB, non-home-based

All correlations are Spearman rho. Correlations in bold indicate statistical significance at p < 0.05.

Category shaded in gray indicates the strongest correlation for the provided sets of measures.

^a^Meeting the aerobic physical activity guideline is defined as ≥150 minutes/week moderate intensity, ≥ 75 minutes/week vigorous intensity, or an equivalent combination of physical activity per week, according to the *Physical Activity Guidelines for Americans*, second edition

^b^n=242 counties

^c^n=240 counties

^d^n=235 counties

When comparing walking trip rates to the prevalence of walking as a top-2 activity and mean frequency of walking in a week, all correlation coefficients were <0.3; only correlations between trips and frequency of walking were significantly different from zero, and the highest observed was 0.23 (Table 3). Similar patterns emerged with walking and bicycling combined: correlations were generally <0.3 but were slightly higher for BRFSS frequency versus prevalence measures (Table 4). One moderate correlation was observed between the any-purpose, weekend, county resident walking and bicycling trip rate and mean frequency of walking and bicycling in a week (rho = 0.30, p < .0001).

**Table 3:** County rank correlations between StreetLight measures of walking trips and BRFSS measures of walking prevalence and frequency.

StreetLight Walking Trips per 1K Population, 2019				BRFSS Physical Activity Measure, 2017 and 2019	
Day	Traveler Type	Purpose	Denominator	Percent of all adults reporting walkingas a top-2 activity in the past month^a^	Mean frequency of walking in a week,from all adults reporting walking as a top-2 activity in the past month^b^
All	Any	All	DP	0.06	**0.20**
Weekdays	Any	All	DP	0.06	**0.20**
Weekends	Any	All	DP	0.06	**0.19**
All	Any	HBO or NHB	DP	0.08	**0.21**
Weekdays	Any	HBO or NHB	DP	0.08	**0.21**
Weekends	Any	HBO or NHB	DP	0.10	**0.21**
All	Residents	All	Residents	0.10	**0.22**
Weekdays	Residents	All	Residents	0.10	**0.22**
Weekends	Residents	All	Residents	0.11	**0.23**

Abbreviations: BRFSS, Behavioral Risk Factor Surveillance System; DP, daytime population; HBO, home-based other; NHB, non-home-based

All correlations are Spearman rho. Correlations in bold indicate statistical significance at p < 0.05.

Category shaded in gray indicates the strongest correlation for the provided sets of measures.

^a^n=239 counties

^b^n=253 counties

**Table 4:** County rank correlations between StreetLight measures of walking and bicycling trips and BRFSS measures of walking and bicycling prevalence and frequency.

StreetLight Walking and Bicycling Trips per 1K Population, 2019				BRFSS Physical Activity Measures, 2017 and 2019	
Day	Traveler Type	Purpose	Denominator	Percent of all adults reporting walking or bicycling as a top-2 activity in the past month	Mean frequency of walking and bicycling in a week, from all adults reporting walking or bicycling as a top-2 activity in the past month
All	Any	All	DP	0.12^a^	**0.26** ^c^
Weekdays	Any	All	DP	0.12^a^	**0.26** ^c^
Weekends	Any	All	DP	0.12^a^	**0.25** ^c^
All	Any	HBO or NHB	DP	**0.16** ^a^	**0.28** ^c^
Weekdays	Any	HBO or NHB	DP	**0.15** ^a^	**0.28** ^c^
Weekends	Any	HBO or NHB	DP	**0.17** ^a^	**0.28** ^c^
All	Residents	All	Residents	**0.15** ^a^	**0.28** ^d^
Weekdays	Residents	All	Residents	**0.15** ^a^	**0.27** ^d^
Weekends	Residents	All	Residents	**0.17** ^b^	**0.30** ^e^

Abbreviations: BRFSS, Behavioral Risk Factor Surveillance System; DP, daytime population; HBO, home-based other; NHB, non-home-based

All correlations are Spearman rho. Correlations in bold indicate statistical significance at p < 0.05.

Category shaded in gray indicates the strongest correlation for the provided sets of measures.

^a^n=239 counties

^b^n=238 counties

^c^n=253 counties

^d^n=252 counties

^e^n=249 counties

When comparing walking trip duration rates per 1,000 people and mean duration of walking, all correlation coefficients were <0.3 except for one moderate correlation: the all-days, any-person, non-commute walking duration rate and mean duration of walking (rho = 0.30, p < .0001, Table 5). Combining walking and bicycling produced similar results with only one moderate correlation: the all-days, any-person, non-commute walking and bicycling duration rate and mean duration of walking and bicycling (rho = 0.31, p < .0001, Table 6), which was the second most strongly correlated measure pair of the 72 StreetLight–BRFSS measure combinations. For both the walking and combined walking and bicycling measures, we observed weak to moderate positive (versus negative) associations only for StreetLight measures restricted to home-based other and not home-based trips.

**Table 5:** County rank correlations between StreetLight measures of walking duration and BRFSS measure of walking duration.

StreetLight Walking Duration per 1K Population, 2019				BRFSS Physical Activity Measure, 2017 and 2019
Day	Traveler Type	Purpose	Denominator	Mean duration of walking in a week, from all adults reporting walking as a top-2 activity in the past month^a^
All	Any	All	DP	**−0.14**
Weekdays	Any	All	DP	**−0.14**
Weekends	Any	All	DP	**−0.14**
All	Any	HBO or NHB	DP	**0.30**
Weekdays	Any	HBO or NHB	DP	**0.28**
Weekends	Any	HBO or NHB	DP	**0.27**
All	Residents	All	Residents	**−0.13**
Weekdays	Residents	All	Residents	**−0.13**
Weekends	Residents	All	Residents	**−0.13**

Abbreviations: BRFSS, Behavioral Risk Factor Surveillance System; DP, daytime population; HBO, home-based other; NHB, non-home-based

All correlations are Spearman rho. Correlations in bold indicate statistical significance at p < 0.05.

Category shaded in gray indicates the strongest correlation for the provided measures.

^a^n=238 counties

**Table 6:** County rank correlations between StreetLight measures of walking and bicycling duration and BRFSS measure of walking and bicycling duration.

StreetLight Walking and Bicycling Duration per 1K Population, 2019				BRFSS Physical Activity Measure, 2017 and 2019
Day	Traveler Type	Purpose	Denominator	Mean duration of walking and bicycling in a week, from all adults reporting walking or bicycling as a top-2 activity in the past month
All	Any	All	DP	**−0.17** ^a^
Weekdays	Any	All	DP	**−0.17** ^a^
Weekends	Any	All	DP	**−0.17** ^a^
All	Any	HBO or NHB	DP	**0.31** ^a^
Weekdays	Any	HBO or NHB	DP	**0.29** ^a^
Weekends	Any	HBO or NHB	DP	**0.27** ^a^
All	Residents	All	Residents	**−0.15** ^b^
Weekdays	Residents	All	Residents	**−0.16** ^b^
Weekends	Residents	All	Residents	**−0.16** ^c^

Abbreviations: BRFSS, Behavioral Risk Factor Surveillance System; DP, daytime population; HBO, home-based other; NHB, non-home-based

All correlations are Spearman rho. Correlations in bold indicate statistical significance at p < 0.05.

Category shaded in gray indicates the strongest correlation for the provided measures.

^a^n=240 counties

^b^n=239 counties

^c^n=238 counties

### Strongest correlation stratified by county characteristics

Across 235 counties, a median of 289 (interquartile range [IQR]: 123) weekend, any-person, non-commute walking and bicycling trips were made per 1,000 people (StreetLight), and a median of 51.0% (IQR: 8.4%) of adults met the aerobic guideline (BRFSS) (Table 7). When stratified by 12 county characteristics with a total of 38 stratified categories, 15 correlation pairs were <0.3, 18 were 0.3 to <0.5, and 5 were ≥0.5. The strong correlations were among medium metro (rho = 0.59, p < .0001), small metro (rho = 0.56, p = .0108), and micropolitan/non-core (rho = 0.50, p = .0059) counties, counties in the least densely populated tertile (rho = 0.52, p < .0001), and counties in the middle tertile of social vulnerability (rho = 0.52, p < .0001). Scatterplots visualize the correlations of counties in each tertile of population density (low: rho = 0.52, middle: rho = 0.34, high: rho = 0.12) (Fig 2).

**Table 7:** County rank correlations between StreetLight measure of walking and bicycling trips^a^ and BRFSS prevalence measure of meeting the aerobic physical activity guideline^b^ by county characteristics.

Characteristic	No. Counties	StreetLight^a^ Median (IQR)	BRFSS^b^ Median (IQR)	Spearman rho	p-value
All	235	289 (123)	51.0 (8.4)	**0.32**	**<.0001**
Census region					
Midwest	51	291 (71)	48.4 (8.2)	−0.16	0.2721
Northeast	54	287 (139)	51.3 (6.4)	0.01	0.9287
South	81	242 (97)	48.6 (7.2)	**0.27**	**0.0132**
West	49	399 (155)	56.8 (6.5)	0.05	0.7527
Urbanicity					
Large central metro	68	315 (145)	50.9 (7.4)	0.20	0.0988
Large fringe metro	64	274 (102)	52.5 (8.1)	0.12	0.3363
Medium metro	54	277 (103)	50.8 (8.3)	**0.59**	**<0.0001**
Small metro	20	308 (152)	51.8 (12.2)	**0.56**	**0.0108**
Micropolitan/Non-core	29	344 (164)	48.4 (7.3)	**0.50**	**0.0059**
Population					
≤ median (≤ 233,464)^c^	118	286 (144)	50.1 (9.8)	**0.39**	**<0.0001**
> median (>233,464)	117	301 (90)	51.8 (8.2)	**0.20**	**0.0285**
Population density^d^					
Tertile 1 (< 219.4)	78	315 (149)	50.4 (10.8)	**0.52**	**<.0001**
Tertile 2 (219.4–1,014.3)	78	277 (100)	52.1 (9.2)	**0.34**	**0.0024**
Tertile 3 (> 1,014.3)	79	299 (109)	50.5 (7.1)	0.12	0.3057
Median age, years					
Tertile 1 (< 36.5)	77	306 (149)	50.1 (8.6)	**0.32**	**0.0043**
Tertile 2 (36.5–40.3)	82	293 (112)	50.3 (8.9)	**0.32**	**0.0034**
Tertile 3 (> 40.3)	76	285 (112)	52.0 (7.6)	**0.36**	**0.0014**
White race					
Tertile 1 (< 57.3%)	78	303 (163)	48.9 (7.2)	**0.25**	**0.0257**
Tertile 2 (57.3% – 79.9%)	77	275 (94)	52.3 (8.7)	**0.42**	**0.0001**
Tertile 3 (> 79.9%)	80	305 (125)	52.4 (9.6)	**0.41**	**0.0001**
Black race					
Tertile 1 (< 3.2%)	78	344 (133)	52.8 (10.5)	**0.35**	**0.0016**
Tertile 2 (3.2% – 12.9%)	78	276 (106)	52.9 (8.2)	**0.23**	**0.0445**
Tertile 3 (> 12.9%)	79	275 (97)	48.3 (6.5)	**0.26**	**0.0185**
Hispanic ethnicity					
Tertile 1 (< 5.4%)	80	276 (120)	49.5 (9.6)	**0.28**	**0.0118**
Tertile 2 (5.4–13.0%)	77	284 (123)	52.3 (8.7)	**0.46**	**<.0001**
Tertile 3 (> 13.0%)	78	319 (134)	50.6 (7.7)	**0.23**	**0.0441**
Some college or more					
Tertile 1 (< 56.6%)	78	276 (112)	46.8 (6.7)	**0.24**	**0.0345**
Tertile 2 (56.6–65.3%)	79	294 (123)	50.2 (6.9)	**0.41**	**0.0002**
Tertile 3 (> 65.3%)	78	308 (131)	55.8 (5.6)	**0.45**	**<.0001**
Below federal poverty level					
Tertile 1 (< 10.6%)	78	296 (111)	53.6 (6.3)	**0.35**	**0.0018**
Tertile 2 (10.6–14.9%)	79	315 (150)	51.6 (7.6)	**0.33**	**0.0031**
Tertile 3 (> 14.9%)	78	276 (111)	46.9 (6.5)	**0.46**	**<.0001**
Median income					
Tertile 1 (<$55,630)	78	275 (112)	46.5 (6.2)	**0.37**	**0.0008**
Tertile 2 ($55,630–68,666)	79	314 (132)	51.8 (7.2)	**0.28**	**0.0124**
Tertile 3 (> $68,666)	78	301 (130)	54.1 (7.2)	**0.40**	**0.0003**
CDC SVI percentile					
Tertile 1 (< 0.33)	79	305 (127)	53.6 (7.2)	**0.34**	**0.0021**
Tertile 2 (0.33–0.62)	74	285 (111)	52.0 (9.4)	**0.52**	**<.0001**
Tertile 3 (> 0.62)	82	284 (148)	46.9 (6.5)	**0.37**	**0.0006**

Abbreviations: ACS, American Community Survey; BRFSS, Behavioral Risk Factor Surveillance System; CDC SVI, Centers for Disease Control and Prevention social vulnerability index; IQR, interquartile range

Correlations in bold indicate statistical significance at p < 0.05.

^a^2019 StreetLight measure of walking and bicycling trips by any person for home-based other and non-home-based purposes on weekends only, per 1K persons of county daytime population (from ACS, 2015–2019)

^b^2017 and 2019 BRFSS measure of percent of all adults who meet the aerobic physical activity guideline (defined as ≥150 minutes/week moderate intensity, ≥ 75 minutes/week vigorous intensity, or an equivalent combination of physical activity per week, according to the *Physical Activity Guidelines for Americans*, second edition)

^c^Median population and all tertiles calculated using ACS data (2015–2019) from the 235-county sample for the BRFSS prevalence measure of meeting the aerobic physical activity guideline

^d^Defined as persons per square mile

**Fig 2:**
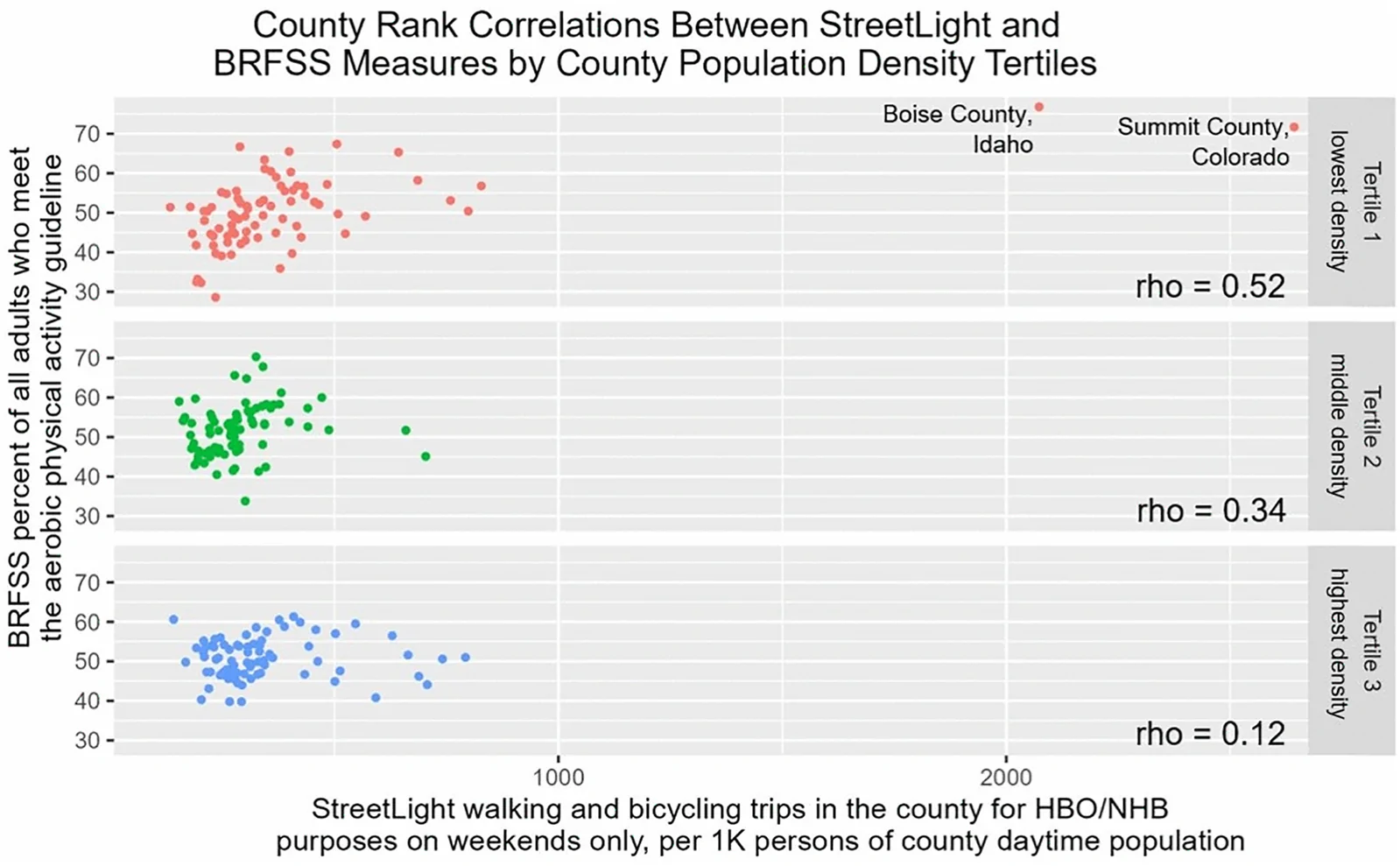
StreetLight walking and bicycling versus BRFSS meeting aerobic guideline^a^ measures, U.S. counties by population density^b^. Abbreviations: BRFSS, Behavioral Risk Factor Surveillance System; HBO, home-based other; NHB, non-home-based. ^a^ Meeting the aerobic physical activity guideline is defined as ≥150 minutes/week moderate intensity, ≥ 75 minutes/week vigorous intensity, or an equivalent combination of physical activity per week, according to the *Physical Activity Guidelines for Americans*, second edition. ^b^ Population density tertiles are calculated using ACS data (2015–2019) from the 235-county sample for the BRFSS prevalence measure of meeting the aerobic physical activity guideline (2017 and 2019). Tertile 1 is < 219.4 persons/mile^2^; tertile 2 is 219.4–1014.3 persons/mile^2^; tertile 3 is > 1014.3 persons/mile^2^.

### Second strongest correlation stratified by county characteristics

Across 240 counties, the median average trip duration accumulated on all-days, any-person, non-commute walking and bicycling trips per 1,000 people (StreetLight) was 848 seconds (approximately 14.1 minutes; IQR: 334 seconds, approximately 5.6 minutes). The median average duration per instance of walking and bicycling in a week, among all adults in the county reporting walking or bicycling as a top-2 activity in the past month (BRFSS) was 28.0 minutes (IQR: 7.9 minutes, Table 8). When stratified by 12 county characteristics with a total of 38 stratified categories, 19 correlation pairs were <0.3, 16 were 0.3 to <0.5, and 3 were ≥0.5. The strong correlations were among medium metro (rho = 0.52, p < .0001) and small metro (rho = 0.53, p = .0160) counties and counties in the middle tertile of social vulnerability (rho = 0.52, p < .0001). Scatterplots visualize the correlations of small metro counties (rho = 0.53) alongside large central metro counties (rho = 0.19) (Fig 3).

**Table 8:** County rank correlations between StreetLight measure of walking and bicycling duration^a^ and BRFSS measure of walking and bicycling duration^b^ by county characteristics.

Characteristic	No. Counties	StreetLight^a^ Median (IQR)	BRFSS^b^ Median (IQR)	Spearman rho	p-value
All	240	14.1 (5.6)	28.0 (7.9)	**0.31**	**<.0001**
Census region					
Midwest	53	14.1 (4.0)	27.3 (6.6)	−0.14	0.3315
Northeast	54	12.7 (5.4)	27.4 (4.3)	0.08	0.5808
South	83	12.3 (4.2)	26.8 (7.2)	**0.29**	**0.0085**
West	50	17.9 (5.5)	36.4 (6.1)	0.10	0.4907
Urbanicity					
Large central metro	68	15.2 (6.4)	28.6 (6.7)	0.19	0.1148
Large fringe metro	65	12.3 (5.9)	28.0 (7.1)	0.23	0.0615
Medium metro	53	13.0 (3.9)	27.5 (9.0)	**0.52**	**<.0001**
Small metro	20	14.6 (5.5)	28.6 (10.9)	**0.53**	**0.0160**
Micropolitan/Non-core	34	14.5 (8.7)	25.4 (16.6)	0.24	0.1789
Population					
≤ median (≤ 233,464)^c^	123	13.3 (6.2)	27.3 (9.5)	**0.27**	**0.0030**
> median (>233,464)	117	14.3 (4.9)	29.0 (7.2)	**0.30**	**0.0009**
Population density^d^					
Tertile 1 (< 219.4)	83	14.3 (7.4)	26.8 (13.7)	**0.39**	**0.0003**
Tertile 2 (219.4–1,014.3)	78	12.6 (4.4)	29.0 (7.5)	**0.35**	**0.0015**
Tertile 3 (> 1,014.3)	79	14.4 (5.8)	28.3 (5.8)	**0.23**	**0.0459**
Median age, years					
Tertile 1 (< 36.5)	75	14.9 (6.6)	28.5 (9.2)	**0.36**	**0.0017**
Tertile 2 (36.5–40.3)	81	14.2 (4.9)	27.8 (7.2)	**0.40**	**0.0003**
Tertile 3 (> 40.3)	84	12.5 (5.1)	28.1 (8.5)	0.18	0.0952
White race					
Tertile 1 (< 57.3%)	76	15.2 (6.4)	27.6 (6.1)	**0.25**	**0.0304**
Tertile 2 (57.3% – 79.9%)	78	12.4 (4.2)	28.2 (8.0)	**0.42**	**0.0001**
Tertile 3 (> 79.9%)	86	14.3 (6.9)	28.2 (11.1)	**0.32**	**0.0025**
Black race					
Tertile 1 (< 3.2%)	84	15.2 (6.3)	30.4 (13.8)	**0.36**	**0.0009**
Tertile 2 (3.2% – 12.9%)	79	12.6 (5.9)	28.5 (6.9)	0.20	0.0774
Tertile 3 (> 12.9%)	77	14.1 (4.4)	27.2 (4.6)	**0.40**	**0.0003**
Hispanic ethnicity					
Tertile 1 (< 5.4%)	84	12.4 (4.7)	26.4 (7.8)	**0.22**	**0.0416**
Tertile 2 (5.4–13.0%)	78	14.3 (5.9)	29.6 (9.8)	**0.48**	**<.0001**
Tertile 3 (> 13.0%)	78	14.9 (5.5)	28.6 (6.9)	0.08	0.4721
Some college or more					
Tertile 1 (< 56.6%)	79	12.3 (4.8)	26.0 (8.5)	0.19	0.0921
Tertile 2 (56.6–65.3%)	80	14.3 (5.1)	27.4 (6.0)	**0.30**	**0.0077**
Tertile 3 (> 65.3%)	81	14.8 (6.3)	31.0 (9.0)	**0.41**	**0.0002**
Below federal poverty level					
Tertile 1 (< 10.6%)	83	13.1 (5.7)	29.7 (8.6)	**0.29**	**0.0068**
Tertile 2 (10.6–14.9%)	80	14.9 (6.0)	27.9 (9.3)	**0.38**	**0.0004**
Tertile 3 (> 14.9%)	77	13.9 (5.4)	27.3 (6.4)	**0.35**	**0.0019**
Median income					
Tertile 1 (<$55,630)	78	13.4 (4.6)	27.0 (9.4)	**0.35**	**0.0016**
Tertile 2 ($55,630–68,666)	82	14.9 (5.5)	27.9 (7.6)	0.17	0.1268
Tertile 3 (> $68,666)	80	13.1 (6.6)	30.0 (8.3)	**0.43**	**<.0001**
CDC SVI percentile					
Tertile 1 (< 0.33)	84	14.3 (6.1)	29.2 (8.7)	**0.29**	**0.0079**
Tertile 2 (0.33–0.62)	73	13.8 (5.0)	28.7 (9.8)	**0.52**	**<.0001**
Tertile 3 (> 0.62)	83	14.2 (6.7)	27.3 (6.4)	0.18	0.0969

Abbreviations: ACS, American Community Survey; BRFSS, Behavioral Risk Factor Surveillance System; CDC SVI, Centers for Disease Control and Prevention social vulnerability index; IQR, interquartile range

Correlations in bold indicate statistical significance at p < 0.05.

^a^2019 StreetLight measure of walking and bicycling trip duration by any person for home-based other and non-home-based purposes on all days of the week, in minutes (provided by StreetLight in seconds), per 1K persons of county daytime population (from ACS, 2015–2019)

^b^2017 and 2019 BRFSS measure of walking and bicycling duration per instance, in minutes, from all adults reporting walking or bicycling as a top-2 activity in the past month

^c^Median population and all tertiles calculated using ACS data (2015–2019) from the 235-county sample for the BRFSS prevalence measure of meeting the aerobic physical activity guideline

^d^Defined as persons per square mile

**Fig 3:**
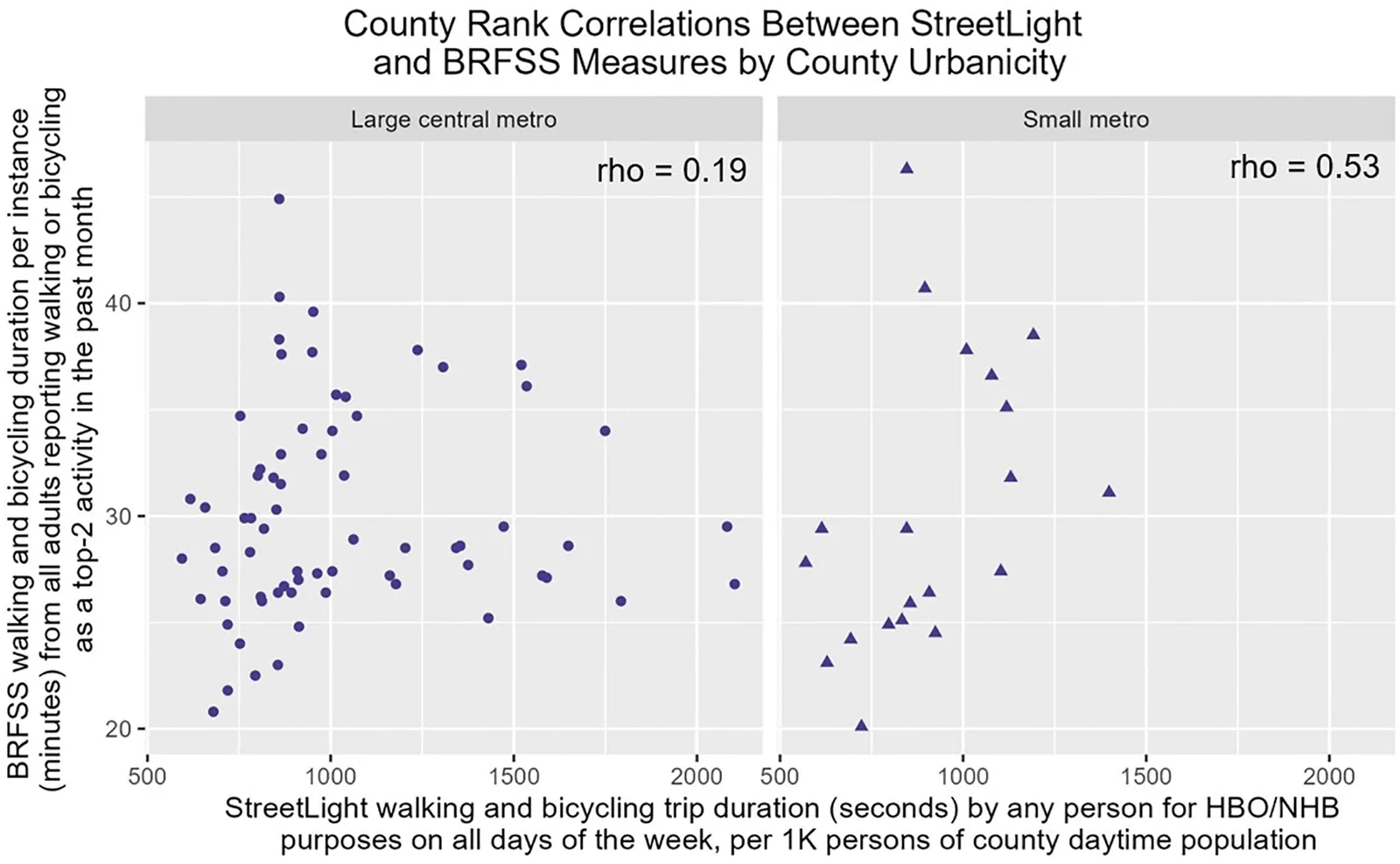
StreetLight versus BRFSS walking and bicycling duration measures, U.S. counties by select urbanicity categories^a^. Abbreviations: BRFSS, Behavioral Risk Factor Surveillance System; HBO, home-based other; NHB, non-home-based. ^a^ From the 2013 National Center for Health Statistics Urban-Rural Classification Scheme.

## Discussion

County rank correlations between passively-collected LBS walking and bicycling measures from StreetLight and self-reported nonoccupational PA measures from BRFSS were mostly low with only a few moderate correlations, suggesting limited convergent validity between these distinct but related data constructs. When the measures with the two strongest correlations overall were stratified by county characteristics, the associations between selected StreetLight–BRFSS measure pairs varied substantially, with strong correlations observed between pairs in medium metro and small metro counties and counties in the middle tertile of social vulnerability. Considering this variation, the convergent validity between LBS measures of walking and bicycling and nonoccupational PA may be context-specific to where the constructs may have more in common. Care may be needed when using LBS measures as indicators of nonoccupational PA at a county level.

Our study is the first to compare StreetLight LBS measures with BRFSS nonoccupational PA measures. A previous study compared county-level StreetLight LBS measures of walking and bicycling with estimates of walking and bicycling to work from ACS. Those results showed mostly moderate to strong correlations between the measures and stronger associations in densely populated, urban counties [8]. In contrast, our study showed mostly low to moderate correlations. Among the two measures with the strongest associations overall, there were stronger associations in less urban counties. Our findings also suggest that narrowing down StreetLight trip purpose (for trip duration measures) might be important for improved correlations with BRFSS nonoccupational PA measures, unlike that observed for StreetLight–ACS correlations for active commuting measures [8].

The contrast in association strengths may be because our study investigates BRFSS nonoccupational PA measures that are more conceptually aligned with leisure-time PA (e.g., by using “exercise” language and by suggesting golf and gardening as examples) with walking and bicycling measures that are only based on respondents’ reported top 2 activities; these measures may be less comparable to StreetLight LBS measures that are typically described and used as transportation constructs (e.g., measuring active transportation such as active commuting) and broadly capture device movement. Depending on their interpretation of the survey questions, BRFSS respondents may or may not include active commuting (i.e., StreetLight home-based work trips) when self-reporting nonoccupational PA. We considered this possibility by creating combination pairs that include StreetLight measures of all purposes and StreetLight measures for non-commute purposes (i.e., home-based other or not home-based trips, which exclude home-based work trips). Another possible contributor to the overall low to moderate StreetLight–BRFSS strength of associations is that people walking and bicycling for leisure may be less likely to carry a mobile device if they walk or bicycle nearby their neighborhood than those who are actively commuting, which would reduce the contribution of leisure activity to StreetLight’s measures and reduce comparability to BRFSS. Moreover, StreetLight does not capture walking or bicycling using stationary equipment, which respondents can report on BRFSS. The well-documented lower prevalence of active transportation in less urban areas may also explain some of our findings, particularly the stronger associations between StreetLight and BRFSS nonoccupational PA measures in less urban counties, where active commuting occurs less frequently [24]. Compared to people in more urban areas, those in less urban areas who are carrying a mobile device while walking or bicycling outdoors (i.e., data collectable by LBS sources like StreetLight) may be more likely engaging in leisure-time versus transportation PA. Theoretically, this would produce stronger associations with measures of leisure or nonoccupational PA from BRFSS.

We largely found that conceptually dissimilar measure pairs were also less correlated. An example is StreetLight trip rates compared with most BRFSS nonoccupational PA prevalence measures. This finding is intuitive because StreetLight trip rates reflect the amount of behavior, whereas BRFSS prevalence estimates reflect the amount of people engaged in the behavior. For instance, a high StreetLight trip rate could represent many trips taken by a small number of people, which would be estimated by BRFSS as a low prevalence. Unsurprisingly, correlations were higher when replacing BRFSS prevalence estimates with mean frequency estimates—the latter of which are conceptually closer to StreetLight trip rates.

Though BRFSS mean duration estimates are also more conceptually comparable to StreetLight duration rates, correlations increased only when limiting StreetLight measures to non-commute trips. This might suggest that differential durations between StreetLight commute (home-based work) trips and non-commute (home-based other and not home-based) trips are sufficiently large enough to alter the direction of county rank correlations with the static BRFSS activity duration metric, producing negative associations (though weak) for trips of all purposes. Previous studies using time diary surveys, questionnaires, and/or GPS data on walking and bicycling time, speed, and distance provide evidence that active transportation trip durations are generally shorter than leisure trip durations, and trip durations to and from work are generally longer than for other active transportation trips [25–28]; a caveat is that non-commute trips can consist of both active transportation and leisure trips with unknown proportions of each.

Though not anticipated due to their conceptual differences, the measure pairs of StreetLight walking and bicycling trip rates and BRFSS prevalence of adults meeting the aerobic guideline were some of the more strongly correlated, particularly with StreetLight weekend, any person, non-commute trips (that plausibly represent more leisure-time PA). This could be because these distinct measures (i.e., StreetLight walking and bicycling trip rates and BRFSS prevalence of adults meeting the aerobic guideline) may be more similar county indicators of nonoccupational aerobic PA levels. This may be especially true in the least densely populated counties (which tend to have less active transportation and active commuting [24]), and small metro, medium metro, and micropolitan/non-core counties—the counties showing strong correlations between these measures.

Notably, two counties stood out in the strongly-correlated least population dense tertile as exemplifying how this measure pair may similarly indicate county levels of aerobic PA: Summit County, Colorado, a micropolitan county, and Boise County, Idaho, a medium metro county. Both had very high daily averages of over 2,000 weekend, non-commute walking and bicycling trips per 1,000 people (StreetLight) and a similarly high prevalence of over 70% of all adult county residents meeting the aerobic guideline (BRFSS). Both are mountainous counties popular for their diverse outdoor recreational activities, with large non-resident tourism populations [29–31]. As mentioned previously, less urban counties may be where these StreetLight non-commute walking and bicycling measures are most similarly ranked with their respective BRFSS nonoccupational PA measures; this is due to the StreetLight measures more closely approximating leisure-time PA in certain circumstances.

Finally, strong correlations (rho = 0.52) were observed for counties in the middle tertile of social vulnerability when the correlations between the two strongest StreetLight–BRFSS measure pairs were stratified by county characteristics. However, it is less clear why middle tertile social vulnerability counties are strongly correlated, in part because the CDC SVI is an index created from 15 different measures in 4 categories: socioeconomic status, household composition and disability, minority status and language, and housing type and transportation [32].

### Implications for physical activity surveillance

This study suggests that LBS data sources may have limited utility for surveillance of nonoccupational or leisure-time PA based on our comparison with these BRFSS constructs. Though some specific county characteristic stratifications showed strong correlations, StreetLight–BRFSS measure pairs were weakly correlated overall with only a few moderate correlations, which would provide limited usefulness as a PA surveillance data source. Among measure pairs with the two strongest associations, less urbanized counties had stronger associations between Streetlight and BRFSS measures; such places may find that the geographic granularity and timeliness of StreetLight LBS data could be beneficial complements to BRFSS when utilizing these specific measure pairs, such as for evaluation purposes. Given previous research, LBS data sources may be more broadly considered for public health surveillance of active commuting behaviors. Regardless, before using emerging PA data sources, including LBS data, researchers and practitioners can acknowledge their limitations and how these may affect any conclusions drawn from the data.

### Limitations

There are limitations to consider in our study. First, BRFSS is weighted at the state level, but our study uses county-level estimates. This could introduce bias because weighted demographic distributions at the state level may not represent county-level demographic distributions, and BRFSS estimates may be associated with these demographic characteristics. Second, StreetLight trip measures and ACS population denominators include people aged <18 years, while BRFSS surveys adults only, so our measure pairs may be less conceptually comparable in counties with younger populations. However, stratifying the measure pairs with the two strongest associations by county-level median age tertiles did not show weaker associations in the youngest tertile. Third, BRFSS self-report measures of walking and bicycling likely underrepresent the total prevalence of walking and bicycling, since respondents are only asked to report their top 2 activities in the past month; only respondents who reported walking or bicycling as a top-2 activity were provided the opportunity to report their frequency and duration in the past month. A respondent who reported other top-2 activities but still walks and/or bicycles would not be represented in prevalence, frequency, or duration measures for walking or bicycling. Likewise, someone who typically walks or bicycles but did not in the past month, at the time of BRFSS survey completion, would not be captured. As with any self-reported survey measure, BRFSS nonoccupational PA measures may also be affected by recall and social desirability biases. Fourth, even though StreetLight captures all LBS trips, it cannot include walkers and bicyclists without smart devices, those who have opted out of location-sharing, or those who regularly leave home without their devices. Our results may not generalize to these individuals [33], though this limitation may be partially countered by StreetLight’s use of bicycle and pedestrian count data as calibration aids. Fifth, because we excluded counties with unstable estimates for that particular BRFSS measure, and StreetLight suppressed some counties’ bicycling trip metrics for county residents, sample sizes for the correlation pairs are slightly different and none include all 298 sampled counties. The suppression of unstable estimates or insufficient sample size also limited our ability to produce standalone bicycling estimates. Sixth, this may introduce selection bias by favoring counties with larger sample sizes (which may be more populous or less rural). However, regarding bias potentially introduced by bicycling suppression of 4 counties at most, reduced county sample sizes favoring larger counties most likely reduced variability within our examined measures; regardless, this ultimately did not affect associations, as our correlation coefficients were very similar even as counties were suppressed. Seventh, our study further assessed the two strongest associated StreetLight–BRFSS measure pairs across county-level sociodemographic and geographic characteristics, but these findings may not be generalizable to other measure pairs that we did not stratify by county characteristics. Eighth, if counties experience substantial discrepancies between resident and daytime populations due to daytime inflow or outflow, and/or if resident and non-resident travelers differed in their trip or PA behaviors, this could variably affect correlations between StreetLight trip measures (which can be restricted to residents or not) and BRFSS nonoccupational PA measures (which are innately restricted to residents). These possible effects could be related to county characteristics that influence resident or daytime populations and resident or non-resident trip or PA behaviors (e.g., urbanicity for business areas, outdoor tourism). Regardless, our study conceptually matched StreetLight measures with their most appropriate corresponding ACS population denominators. Ninth, this exploration of convergent validity between StreetLight and BRFSS measures was a county-level ecological analysis that may or may not reflect individual-level data, and the correlations from this study are not suitable for drawing conclusions about individuals. Finally, the data sources we used were temporally mismatched (i.e., 2019 StreetLight, 2017 and 2019 BRFSS, 2015−2019 ACS, 2018 SVI data), which could have impacted the precision and thus comparability of estimates. However, we prioritized including counties with enough sample size and using available data to explore convergent validity within a timespan with more stable physical activity or human movement patterns (i.e., before COVID-19).

### Future research

To add to our knowledge of how LBS data compare to traditional public health surveillance systems for PA, follow-up research could investigate how the sociodemographic breakdown of LBS walking and bicycling trip data, inferred by a device’s home block group, compares to the sociodemographic characteristics of BRFSS respondents who report walking and bicycling as top-2 activities. Further study with other traditional surveillance systems may also be necessary to clarify or confirm convergent validity between LBS data and self-reported survey data of various PA domains, such as leisure-time or transportation PA. Additional research may also further explore associations in the least populous areas, which may have been disproportionately impacted by data suppression in our research.

## Conclusions

We observed weak to moderate associations between county-level passively-collected StreetLight LBS walking and bicycling measures and self-reported BRFSS nonoccupational PA measures; this suggests limited convergent validity between these related but distinct data sources. Generally, associations were higher for conceptually similar measure pairs and trip durations limited to non-commute purposes. When the two most strongly correlated measure pairs were stratified by county characteristics, stronger associations were observed for less urban counties and counties of medium social vulnerability; this suggests possible interchangeability and thus a potential use for these data in certain types of counties, where the constructs may have more in common. LBS data may also be promising for representing other domains of PA, such as active transportation.

## Supporting information

S1 Dataset. 2019 StreetLight county metrics.
(XLS)
